# Quantification des besoins en intrants antipaludiques: contribution à l'actualisation des hypothèses pour la quantification des intrants de prise en charge des cas de paludisme grave en République Démocratique du Congo

**DOI:** 10.11604/pamj.2015.20.94.4514

**Published:** 2015-02-03

**Authors:** Joris Losimba Likwela, John Otshudiema Otokoye

**Affiliations:** 1 Programme National de Lutte Contre le Paludisme (PNLP), Département de Santé Publique à la Faculté de Médecine et de Pharmacie de l'Université de Kisangani, République Démocratique du Congo (RDC); 2 Initiative Présidentielle Américaine de Lutte contre le Paludisme (PMI), Agence Américaine pour le Développement International (USAID), République Démocratique du Congo (RDC)

**Keywords:** intrants antipaludiques, paludisme grave, DRC, intrants, antimalarial inputs, severe malaria, DRC, inputs

## Abstract

Les formes graves de paludisme à *Plasmodium falciparum* sont une cause majeure de décès des enfants de moins de 5 ans en Afrique Sub-saharienne. Un traitement rapide dépend de la disponibilité de médicaments appropriés au niveau des points de prestation de service. La fréquence des ruptures de stock des commodités antipaludiques, en particuliers celles utilisées pour le paludisme grave, avait nécessité une mise à jour des hypothèses de quantification. Les données issues de la collecte de routine du PNLP de 2007 à 2012 ont été comparées à celles rapportés par d'autres pays africains et utilisées pour orienter les discussions au cours d'un atelier organisé par le PNLP et ses partenaires techniques et financiers afin de dégager un consensus national. La proportion des cas de paludisme rapportés comme grave en RDC est resté autour d'une médiane de 7% avec un domaine de variation de 6 à 9%. Hormis la proportion rapportée au Kenya (2%), les pays africains ont rapporté une proportion de cas grave variant entre 5 et 7%. Il apparaît que la proportion de 1% précédemment utilisée pour la quantification en RDC a été sous-estimée dans le contexte de la gestion des cas graves sur terrain. Un consensus s'est dégagé autour de la proportion de 5% étant entendu que des efforts de renforcement des capacités seraient déployés afin d'améliorer le diagnostic au niveau des points de prestation des services.

## Introduction

Le nombre de cas et de décès de paludisme a été estimé à 207 millions et 627000 respectivement en 2012. La plupart des décès estimés (90%) ont eu lieu en Afrique subsaharienne essentiellement parmi les enfants de moins de 5 ans (77%) [1]. Les formes graves sont dues au *Plasmodium falciparum*, espèce la plus fréquente en République Démocratique du Congo (RDC), et se développent très rapidement chez l'enfant, généralement suite à un retard dans la prise en charge des cas simples [1, 2]. Un traitement rapide dépend de la disponibilité de médicaments appropriés pour traiter le paludisme grave via un approvisionnement ininterrompu basé sur une quantification rationnelle. Le financement international s'est considérable accru ces dernières années, dont une des composantes majeures porte sur l'approvisionnement en commodités antipaludiques. Toutefois, en raison, entre autres, de difficultés dans la quantification des besoins pour traiter les patients pour un paludisme sévère, les médicaments nécessaires sont souvent indisponibles en quantités suffisantes [2, 3]. En RDC, la proportion des cas de paludisme grave utilisée depuis plusieurs années est de 1% de l'ensemble des cas de paludisme. Cependant, la fréquence des ruptures de stock des traitements pour le paludisme grave observée dans les formations sanitaires laisse supposer une insuffisance dans les approvisionnements. A l'occasion de la révision du plan stratégique national de lutte contre le paludisme et à la faveur de la dotation du Fonds Mondial dans le cadre du financement intermédiaire pour le comblement des carence identifiées dans les subventions en cours, une réévaluation de la proportion des cas de paludisme grave utilisée pour la quantification des besoins en intrants, par la méthode basée sur la morbidité, s'est avérée indispensable. Nous présentons ici la démarche utilisée pour estimer la fréquence des cas de paludisme prise en charge comme cas grave dans les services de santé et qui consomment les intrants destinés à la prise en charge des cas de paludisme grave afin d'ajuster la base quantification des antipaludiques en RDC.

## Méthodes

Les données issues de la collecte de routine du PNLP ont été analysées afin de déterminer la proportion de cas rapportés comme sévères parmi les cas prise en charge comme cas de paludisme dans les services de santé intégrés au système de santé. Ces données ont été comparées à celles des autres PNLP d'Afrique sub-saharienne obtenue au moyen d'une revue de littérature réalisée par Shretta R et al [2]. Les données ainsi colligées ont servi de base de discussion avec les experts du PNLP et ses partenaires techniques et financiers réunis en atelier afin d'obtenir un consensus sur la position à adopter pour la quantification des intrants de prise en charge du paludisme sévère en RDC.

## Résultats

**Figure 1:**
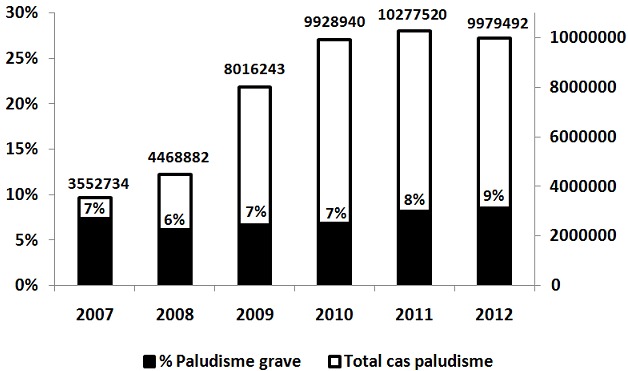
Évolution des cas de paludisme rapportés et de la proportion des cas graves de 2007 à 2012 en République Démocratique du Congo

On observe une augmentation des cas de paludisme reçus dans les services de santé ces 6 dernières années. La proportion des cas de paludisme rapportés comme grave par contre, est resté autour d'une médiane de 7% avec un domaine de variation de 6 à 9% (Figure 1). Hormis la proportion rapportée au Kenya, tous les pays sélectionnés par Shretta R et al ont rapporté une proportion de cas grave variant entre 5 et 7% (Tableau 1).

**Tableau 1:** Prévalence des cas simples et proportion des cas graves pour différents pays africains sélectionnés

Pays	Nombre annuel de cas de paludisme simple	Proportion de cas de paludisme grave
Uganda*	10.203.972	5%
Tanzanie*	11.644.345	6%
Kenya*	7.960.000	2%
Sud Soudan*	4.339.487	7%
Malawi*	4.204.468	5%
RDC**	8.305.811	7%

* Source: Shretta R et al

** Médiane des données PNLP-RDC de 2007 à 2012

## Discussion

Parmi les deux approches utilisées pour la quantification des intrants, celle basée sur la consommation et celle basée sur la morbidité, la RDC utilise présentement la seconde. Cependant, au moment des soumissions précédentes à la subvention du Fonds mondial de lutte contre le sida, la tuberculose et le paludisme, l'insuffisance des données avait conduit le pays à utiliser les estimations réalisées sur base des données publiées et non publiées par des groupes tels que Malaria Risk Across Africa (MARA) et Child Health Epidemiology Reference Group (CHERG) [4]. Ces estimations avaient conduit à un consensus sur 500 millions d’épisodes cliniques parmi lesquels 2 à 3 millions de cas sévères [4]. Le pays avait tablé sur 1% de cas graves attendus sur l'ensemble des cas. Les données recueillies en routine par le PNLP de la RDC et des cinq autres pays limitrophes ont montré que cette proportion était sous-estimée dans le contexte de la prise en charge des cas sur terrain. Les erreurs de classification et le sous équipement des formations sanitaires peuvent avoir conduit à une surestimation des cas de paludisme graves incluant dans cette catégorie des cas soit simples, soit des maladies fébriles sévères d'autre origine [5, 6]. Cependant, il faut relever que c'est sous cette classification que les prestataires prennent en charge les malades et donc consomment les intrants de prise en charge des cas grave [7]. En conséquence,, il importe de poursuivre la mise à l’échelle des tests de diagnostic rapide et des efforts de renforcement des capacités des prestataires au moyen de la formation des soignants sur les lieux de prestation suivi de supervisions formatives avec pour finalité de réduire progressivement la proportion des cas de paludisme grave avec notamment un diagnostic correct et une prise en charge appropriée des cas simples [8, 9].

## Conclusion

Il est apparu donc une nécessité de revoir à la hausse la proportion à utiliser pour la quantification afin d'assurer un approvisionnement cohérent avec la consommation actuelle au niveau des lieux de prestation des services. Un consensus s'est dégagé autour de la proportion de 5% étant entendu que des efforts de renforcement des capacités étaient en cours afin d'améliorer le diagnostic au niveau des points de prestation des services.
